# Medical Miscellanies

**Published:** 1817-02

**Authors:** 


### PART IV.

### MEDICAL MISCELLANIES.

### THE PORTE FEUILLE, No. II.

OR,

DEPOT FOR DETACHED FACTS AND INTERESTING PHÆNOMENA, OCCURRING IN THE PRACTICE OF THE FRIENDS OF THE MEDICO-CHIRURGICAL JOURNAL.

"Ore trahit quodcunque potest atque addit acervo."

#### *Curious Abdominal Disease.*

A child, 3 years of age, died, and was opened by the writer. It had been ill for more than twelve months, with a progressively increasing abdomen, which was considered ascites. On striking the abdomen, in the dead subject, the writer remarked, that he did not concieve the disease to be dropsy (he had not seen the little patient alive), but rather tympanitis. On opening the abdomen, a great quantity of air rushed out. On laying back the parietes, a bag appeared adhering firmly to the liver, spleen, and diaphragm, and reaching down to the pubis. It was adherent on the sides and back; but on raising it at the bottom, the rectum was seen decending from it into the pelvis. All within it felt a semi-solid mass. It was cut into, when the various convolutions of the intestines were found winding through a mass of mesenteric disease. Some of the mesenteric glands were as large as nutmegs. The intestines in every part of their track were fixed in this diseased structure, and could not possibly have had any peristaltic motion. They maintained their place, and even their calibre, when the morbid mass was cut through in all directions. They were partly filled with liquid fæcal matter, and partly with air. The liver was studded throughout with tubercles. Was the bag a morbid secretion of coagulable lymph?

### *Anomalous Disease.*

On the 4th of October, 1816, Jane Birn, a delicate child, about 6 years old, was seized with vomiting and purging. On the 8th she was visited by a physician. The vomiting had continued from the first seizure, but there had been no stool subsequent to the 4th. The matter ejected was considerable, and in colour resembling coffee grounds. Rhubarb, Godfrey's Cordial, and Daffy's Elixir, had been given at intervals. The tongue was thickly coated, brown in the centre; the edges and apex of a fiery red. Pulse at the wrist 130; at the carotid and temporal arteries about 100. Countenance flushed; delirium with screaming; the eye-lids constantly closed; but on exposing the eye, the pupils easily contracted and dilated. Heat of body little above the natural standard; skin soft; no tension of the abdomen or evidence of pain on pressure.

Five grains of calomel with the third of a grain of opium directly; and a table spoonfull of a mixture of infus. sennæ Tr. of jalap and manna every two hours, were directed. To be put into a warm bath at 94° for ten minutes.

9th. Vomiting unabated; all the medicine rejected; no stool; pulse the same Repeat the warm bath; apply a blister to the scrob. cord.; six drachms of sulphate of soda in twelve ounces of gruel, to be given as an enema every four hours.

10th. At nine in the morning, four injections had been given; from each of the last two there was a copious discharge of dark and highly offensive fæculent and other matter. Vomiting incessant; muttering delirium; features shrunken; pulse incalculable; skin generally cold; forehead and breast covered with a cold moisture; tongue nearly black; inside of the lips and teeth covered with black sordes. Three minnims of Tr. opii in a tea-spoonful of cinnamon water every four hours.

3. P. M. Five doses of opium taken; no vomiting since 10 o'clock; some tea and gruel had been taken and retained; sleeping calmly; pulse more distinct; opium to be suspended, and resumed if the vomiting returns. Two grains of calomel, and eight of compound powder of scammony, every hour, until the bowels are freely relieved.

11th. At 12 this day, Eight powders had been taken and retained; as also portions of thin drink. No stool; pulse 112; skin warm; no delirium; had slept comfortably during the night. While an injection was preparing, a copious evacuation took place. Should there not be five or six motions by bed time, to throw up an enema.

12th. There have been eight copious stools; the five first, dark and offensive; but no hardened fæces. The three latter stools of a healthy appearance; pulse 90; skin warm and moist; tongue nearly clean; appetite returning. Ten grains of jalap and two of compound cinnamon powder twice a day.

14th. Except a sense of debility, no complaint. Continue the powders for several days.

*Query.* Was this inflammation of the stomach rather than affection of the cerebrum? L.

We are inclined to think that the affection of the encephalon was sympathetic and only functional, while the seat and origin of the disease was in the abdomen. Editors.

A lady, turned of 30, and for many years subject to an obstinate cutaneous eruption, which baffled every remedy, experienced the following curious phænomenon. When lying straight along in bed, whether on the back or sides, she felt no pain or uneasiness; but the moment she drew up her legs, so as to bend the hips and knee joints, she was seized with a pain in the head which continued till she resumed her former posture.

H. F. a labouring man, about 40 years of age, received a blow by a maul on the left cheek, a little below the eye, about eighteen months ago. Some small red spots soon appeared on the part, which afterwards degenerated into an irritable sore, which continued open more than twelve months. A few months after the receipt of the injury he became affected with pain on the crown of the head, which was so violent as nearly to deprive him of his reason. He was cupped, blistered repeatedly, purged, &c. without much relief. He was then put on a mercurial course, and, when salivation had been sometime established, he obtained a temporary relief. The pain, however, returned as the influence of the mercury subsided. The same medicine was repeatedly had recourse to, and with the same results. It was now obseived, that the right eye had an obliquity towards the nose, and he found that the sight was impaired. He was recommended to have the smoak of tobacco blown into his ears by some old woman; and certain it was that the pain, about this time, was so signally mitigated, that he ascribed the cure entirely to this remedy.

Shortly after this event, he began to lose sensation in the right half of the scalp; in the cheek of that side, and also in the right arm. The sight of the right eye was gone, and he could bear the eye to be rubbed and handled, in the roughest manner, without the least inconvenience. The pain of the head was now gone also, and he considered himself as fortunate in the change from continued suffering to his present state of insensibility.

The wound of the cheek healed about the time that the pain was mitigated, by (as he thought) the tobacco fumes.

---

### MISCELLANEOUS.

Quicquid agunt Homines.

*To the Editors of the Medico-Chirurgical Journal & Review.*

GENTLEMEN,

The progress of vaccination is at all times an interesting subject to Englishmen, as the discovery is so peculiarly our own; I therefore, send for insertion in your Journal, an account of the favourable state of vaccination in the Island of Java, obtained from the highest authority.

I am, &c.

B. W. COLEY,

Member of the Royal College of Surgeons, London; a Surgeon in the Royal Navy, and resident Practitioner at Cheltenham.

*Cheltenham, Dec.* 10, 1816.

It must be as satisfactory to the advocates of humanity in general, as gratifying to the distinguished discoverer of vaccination, to learn, that during the short period of the British administration in Java, a system has been there adopted, which, by engrafting the vaccine establishment on the native institutions of the country, affords, to a population of nearly five millions, the blessings of this invaluable discovery; which promises to outlive our own authority in that quarter of the Globe. In arranging the revenue establishment of the country, particular portions of land attached to each village, have been set apart for the support of the vaccine establishment; and a certain number of native vaccinators have been selected in each district. These vaccinators are under the immediate superintendance of European surgeons, who also derive their emoluments from the revenue of the lands thus appropriated; and the British Government has foregone its right, for ever ,in favour of this institution. The amount is about one per cent. on the gross revenue of the country; and as a mark of respect and veneration to Dr. Jenner, the lands, or sawahs, as they are called, have been designated *Sawah Jennerian*, and as such, are recognized in all the rent rolls of the country; the word *Jennerian*, harmonizing in a peculiar manner with the language of the country, and is, by this means, rendered an integral part of it. By this arrangement, every one must admire the classic taste of the governor of Java, in thus immortalizing the name of Jenner, and the ability displayed in establishing the institution on so novel and durable a basis.

Vaccination had been introduced into the eastern islands in the time of the Dutch, but no general plan had ever been adopted for diffusing its great advantages throughout the country, or for rendering it completely permanent. Indeed, it was some time before any effectual measures to this effect were taken by the British, and it was only on the int oduction of an entire new system of government, that it could possibly be attended with all the desired success. While it was the policy of the Dutch Government to keep down the strength, and even the resources of the country, lest it should become too powerful for the governing power, much could not be expected; but when the rights of property were more fully established in this extensive island, and the government was no longer jealous of its own subjects, it became anxious further to promote their welfare, riches, and power; and the time seemed to have arrived, when it would have been unpardonable in the local authority, not to have diffused the system of vaccination among them. The ravages made by the small pox during the two centuries in which this valuable colony has been under European authority, have been dreadful, and the native inhabitants, among whom the new arrangements are decidedly popular, have given an earnest, that they are competent to appreciate their advantages and to uphold their continuance, under whatever political circumstances the island may be placed. The chief native authority of the island, generally termed by Europeans the *Emperor*, caused his family to be vaccinated in public; and on a recent occasion held his youngest child in his arms, while the operation was performed, as an encouragement to his subjects, who have already been vaccinated to the amount of many thousands; and when the late accounts left Java, the establishment was in full activity.

---

### *Charge of Plagiarism against the Medical and Physical Journal.*

"Plagiary, *author-theft;* or the practice of purloining other people's works, and putting them off as a man's own."—*Rees's Cycloped.*

THE *Collectanea Medica* division of the London Medical and Physical Journal for January, 1817, begins thus:—"*We* have so much continental matter, and some of it has been so long on *our* shelf, that we must dedicate to *it and our remarks* the division of Collectanea for this month." "The *first* is a communication from Dr. Von Embden, &c." This paper is marked with inverted commas. The Editors then proceed:—"The *next* paper shews, &c;" and this paper also is marked with inverted commas, as the first; and *without any reference at the end.* Now, we ask every man of *common plain sense,* whether he would not consider both these papers as part of so much "continental matter," which had been "*so long on the shelf,*" and either collected by the Editors or contributed by their friends?—Yet, strange as it may appear, the *second* paper, consisting of seven closely printed pages, was *tr nslated and compiled* from the French language, *one short month bfore,* for the *Medico-Chirurgical Journal,* by one of its literary constituents; and lo! it is published, without acknowledgment, in the succeeding No. of the Medical and Physical Journal, as matter that had been "so long on *their* shelf!"— If this be not a *virtual* plagiary, then we acknowledge that we know not what the term means.

But this is not all. The judgement of the *Medico-Chirurgical* writers is impugned, and *malicious* motives imputed to them by the very men who *pilfer* their labours! "All this *absurdity*," says the Med. Phys. Journal, "might be passed over with a smile, were it not for a MALICIOUS GRIN at a most respectable character, and conveyed to a whole nation." But let us ask, why the Medical and Physical Editors transcribed seven pages of "*all this absurdity*" into their work, when they had "so much continental matter" on *their* shelf? — The *malicious grin*, as this elegant Journal styles it, means our indignant reflection on the iniquitous conduct of Dr. Rush, if the accusation preferred in the French Dictionary of Medical Sciences were correct. This we repeat again and again. Talent, rank, and reputation are not, in our eyes, any palliation of flagrant perjury, and venal tergiversation. The *accusation* comes not from us; but an expression of abhorrence of the crime, if committed.

If the Medico-Chirurgical Journal, thus injured and insulted, declines retaliation, it is not from fear of crossing bayonets with even a more formidable enemy. True it is, that it cannot cover its *front* with triple or quadruple lines of ancestral skeletons; nor can it hide beneath the *painted sepulchres* of its *departed* forefathers, the degeneracy of their progeny. Yet it has industry enough to render it independent of others; and it has courage enough to protect its property, and check the predatory incursions of those adventurers who live by plundering their neighbours!

THE MEDICO-CHIRURGICAL JOURNAL.

---

### *Cases of Cardiac Disease from the French of M. Merat.*

CASE 1. *Cardi-pericardiac Adhesion. Active Dilatation of the left Ventricle, &c.*—Louis Aug. D. ætat. 12, had been always valetudinary; yet he was at the same time studious, and fond of the amusements of his age. For some time past his respiration was tight, on which symptom fever supervened, with pain in the left side of the thorax, and sanguineous expectoration. These continued about a month, when he recovered so far as to resume his accustomed pursuits; but a short respiration and frequency of pulse remained. Six months afterwards, he experienced a slight relapse: after which the pain of side, and shortness of breath were aggravated; yet the lad became in some degree accustomed to his complaints, which did not prevent him from play and employment. Three months after this relapse, he again fell ill, when M. Merat found him pale, except the lips, which were like violets. The heart beat furiously and tumultuously; the pulse partook of the same commotion; yet he had neither cough nor pain in the side; and his tongue was clean. For two or three days he vomited every thing which he took. Some leeches were applied to the region of the heart, and some diluent antispasmodic drink prescribed. On the third day, the breathing became more difficult; the patient took in deep inspirations from time to time, coughed a little, and expectorated purulent matter. When asleep we could see the whole breast, and part of the abdomen heave with the cardiac pulsation, The subclavian and carotid arteries also beat furiously: the pulse exceedingly quick. On the sixth day, the respiration became very difficult. The next day, death closed the scene.

*Sectio Cadaveris.* Great general emaciation. The lungs sound and non-adherent. The heart occupied a great portion of the anterior region of the chest. It was at least thrice its natural size; the left ventricle was more enlarged than any other cavity of the heart. The valves both of the heart and arteries were sound. The pericardium adhered every where firmly to the heart by means of cellular membrane, apparently of not long standing. It was red, and bore evident marks of inflammation. The origins of the great arteries were natural. The abdominal viscera were sound.

It is probable, that the cardiac dilatation, in this case, was of very remote date; perhaps congenital. Mons. Merat thinks, that the pericarditis was the immediate cause of the boy's death; as the organic affection of the heart itself was not likely to produce that event.

CASE 2. *Passive Aneurism of the right Cavities of the Heart.*— Louis M———, porcelaine painter, of a debauched character, and having frequently experienced venereal attacks, perceived, in the spring of 1806, a sense of oppression in the chest, which was increased by walking. There was no *decubitus difficilis;* but his sleep was broken; he had cough and fœtid expectoration. About a fortnight afterwards, he experienced dyspnœa and palpitations of the heart. These came on in paroxysms, especially in the night; but diminished and finally ceased when expectoration was completely established. These paroxysms became gradually aggravated, especially the cough, and oppression of the chest. The legs now swelled; he had *decubitus difficilis* on the right side and back. The pulsations of the heart were at this time lively, tumultuous, and weak. They could scarcely be felt during inspiration and expiration. On placing the hand on the region of the heart, a kind of trembling sensation was felt ascending along the middle of the sternum. During the next ten weeks, all these symptoms were exasperated; the pulse became smaller; more frequent; more irregular. The patient was obliged to rest always in the sitting posture. Mortuus est.

*Sectio Cadaveris.* The body in general œdematous; the face and neck bloated. The lungs were filled with a yellowish serum, and presented when cut, shreds of false membrane organized, and resulting from chronic inflammation. The heart was double its natural size. The right auricle was very large, and filled with coagulated blood. Its parietes were attenuated. The auriculo-ventricular opening was præternaturally large. The right ventricle was enlarged, and its parietes thin. The left ventricle was natural, as were the origins of the great vessels.

The feeble stroke of the heart indicated passive aneurism in this case; and the tumultuous movement under the sternum indicated also, that the dilatation was seated in the left cavities of the heart.

CASE 3. *Rupture of the Chordæ Tendineæ of the left Ventricle.*—F. F. ætat. 18, a factor, had had a scorbutic affection in his youth. About 12 years of age, he felt, for the fisrt time, some painful dragging sensations in the epigastric and umbilical regions, which could only be relieved by flexion forward of the body. These went off, after some time, and he enjoyed pretty good health, till the month of August 1805, when he was seized with a violent pain about the præcordia, accompanied by an overwhelming sense of oppression. He coughed. Spat up blood. The cough continues. During a month he wasted much; became overburthened, and oppressed. His hands were burning; his bowels irregular. The beats of the heart were very strong, and very frequent; but the pulse was not in a similar state. About this time, the patient was suddenly seized, after a slight repast, with a kind of convulsion. He tossed about violently; and seemed to have some extraordinary internal affection. He said his sufferings were dreadful, and begged to be dispatched! He could not rest a moment in one posture. The pulse presented great variety; at one time extremely quick but small; anon, regular. He expired after two hours indescribable sufferings.

*Sectio Cadaveris* The pericardium was much distended, and contained about a pint and a half of serum. The heart was swimming in this fluid; and appeared larger than natural. The mitral valve appeared cut off, (déchiqueté) from a number of tendinous filaments seen loose on its border. These were portions of the tendons of the carneæ columnæ, the whole of which (the tendons) excepting perhaps about seven or eight, were torn. The calibre of the aorta was a little straitened.

We cannot compliment M. Merat on the shrewdness of his observations on this case. He thinks the state of languor and malaise which during a month preceded the accident, extended its debilitating influence to the *carneæ columnæ* of the heart—"et que *leur* rupture instantanée fut le resultat de cette action affaiblissante." It is difficult to conceive how any mind in a state of sanity, could weave such a tissue of absurdity and contradiction as is here presented! But one moment before, he tells us that it was the *chordæ tendineæ* which were ruptured, and not the carneæ columnæ! Who would have conceived that the *debility* of a muscle would cause the rupture of its tendon? "Ou pourrait croire que l'hydro-pericarde qu'on observa chez ce jeune homme, a pu contribué aussi a la debitile *musculaire* de cet organe." This is adding blunder to blunder!

### *Efficacy of Mercury in Congestive Typhus.*

An old robust gentleman was seized by typhus, which assumed the inflammatory character; his wife, an extremely delicate woman, who attended a great deal upon him, was in a few days infected with the fever; but in her it put on the congestive form. She was from the first completely overpowered; her face grew extremely pale, and had a dejected expression; her tongue was white, but moist; she felt her head uneasy and heavy, but her mind was not disordered; her pulse became weak and irregular; and her skin relaxed and cool. She principally complained of an extreme load and oppression about the epigastric and right hypochondriac regions, together with great loss of strength. On the bowels being freely opened with calomel and jalap, the stools appeared of the colour and consistence of tar. Finding that some relief was experienced, the purgative and alterative plan was continued for about four days, at which time ptyalism occurred; and it was curious to remark how the excitement emerged with the mercurial action, and how the indications of visceral congestion receded. Yet still there was a tendency to relapse in this case, which required to be counteracted by the regular exhibition of purgatives. In this lady the liver seemed to be the principal seat of congestion, and partly on this account, and her extreme delicacy, the cure was chiefly confided to aperients and mercury; though the warm bath was occasionally used, and a moderate portion of diffussible stimulus allowed, whenever she felt faint from the evacuations.—*Armstrong's Illustrations of Typhus.*

*Analogy between the Kidnies and Testicles.*—From the original propinquity of the kidnies to the testicles; from the renal and spermatic artery so often coming out of the aorta in one trunk; from the renal plexus of nerves sending downwards to the testicles one or more fillets; and from the intimate connection in structure between the urinary and genital apparatus, may we not legitimately presume, that there is also between them a connection in function? And, since the genital is a primary or fundamental function in the animal œconomy, that the urinary secretion is subservient to the business of generation? Is not this conjecture supported by the trim of the generative function being so proportional to the urinary secretion; by those substances which excite urinary secretion stirring up at the same time the generative appetites and powers; by morbidity of urinary secretion being always accompanied by morbidity of the generative function; witness the concomitant want of urea and of sexual desire in diabetes: and I had almost subjoined, is not the above conjecture supported by the long strings of rules, the result, without doubt, of more or less experience which have been so confidently laid down by old medical authors for ascertaining the procreative powers from the qualities of the urine?—*Mr. Cross, in the Annals of Philosophy.*

LECTURES ON THE EYE AND EAR.—Mr. STEVENSON purposes commencing his annual Course of Lectures on the Anatomy, Physiology, and Pathology, of the Eye and Ear, early in March.

In a series of twenty-four Lectures will be given a full anatomical description and demonstration of every part of the complex and minute structure of the organs of sight and hearing ; an explanation of their respective functions in a state of health, and an elucidation of the history, symptoms, and best mode of treating their various diseases : including an account of all the requisite surgical operations, and the most approved method of performing them.—Particulars may be had by application to Mr. Stevenson, at his house, 105, Great Russel Street, Bloomsbury Square.

Dr. MERRIMAN and Dr. LEY will recommence their Course of Lectures on the Theory and Practice of Midwifery and the Diseases of Women and Children, at the Middlesex Hospital, on Monday, February 17, at half past ten o'clock.

Mr. COPLAND HUTCHISON, late Surgeon to the Royal Naval Hospital at Deal, &c. has in the Press, and shortly to be published, "Some farther Observations on the subject of the Proper Period for Amputating in Gun-Shot Wounds, accompanied by the Official Reports of the Surgeons employed in his Majesty's ships and vessels at the late Battle before Algiers."

An Inquiry into the Effects of Spirituous Liquors upon the Physical and Moral Faculties of Man, and their Influence upon the Happiness of Society, is in the Press, and will speedily be published.

